# Comparative sequence analysis of patient-matched primary colorectal cancer, metastatic, and recurrent metastatic tumors after adjuvant FOLFOX chemotherapy

**DOI:** 10.1186/s12885-019-5479-6

**Published:** 2019-03-21

**Authors:** Kazuaki Harada, Wataru Okamoto, Sachiyo Mimaki, Yasuyuki Kawamoto, Hideaki Bando, Riu Yamashita, Satoshi Yuki, Takayuki Yoshino, Yoshito Komatsu, Atsushi Ohtsu, Naoya Sakamoto, Katsuya Tsuchihara

**Affiliations:** 1 Department of Gastroenterology and Hepatology, Graduate School of Medicine, Hokkaido University, Kita 15, Nishi 7, Kita-ku, Sapporo, Hokkaido 060-8638 Japan; 2 Biobank Translational Research Support Section, Translational Research Management Division, Clinical Research Support Office, National Cancer Center Hospital East, 6-5-1 Kashiwanoha, Kashiwa, Chiba 277-8577 Japan; 3 Division of Translational Informatics, Exploratory Oncology Research and Clinical Trial Center, National Cancer Center, 6-5-1 Kashiwanoha, Kashiwa, Chiba 277-8577 Japan; 4 Hokkaido University Hospital Cancer Center, Kita 14, Nishi 5, Kita-ku, Sapporo, Hokkaido 060-8648 Japan; 5 Department of Drug Therapy, Aichi Cancer Center, 1-1 Kanokoden, chikusa-ku, Nagoya, Aichi 464-8681 Japan; 6 Department of Gastrointestinal Oncology, National Cancer Center Hospital East, 6-5-1 Kashiwanoha, Kashiwa, Chiba 277-8577 Japan; 7 National Cancer Center Hospital East, 6-5-1 Kashiwanoha, Kashiwa, Chiba 277-8577 Japan

**Keywords:** Colorectal cancer, Whole exome sequencing, Mutagenicity, Adjuvant chemotherapy, Chemo-resistance

## Abstract

**Background:**

In the era of genome-guided personalized cancer treatment, we must understand chemotherapy-induced genomic changes in tumors. This study evaluated whether adjuvant FOLFOX chemotherapy modifies the mutational profile of recurrent colorectal cancer (CRC).

**Methods:**

Whole exome sequencing was performed on samples from primary CRC tumors, untreated metastatic tumors, and recurrent tumors following adjuvant FOLFOX chemotherapy. The samples were resected from four patients.

**Results:**

The number of mutations or the mutation spectrum in individual patients was nearly identical. Copy number variants persisted regardless of FOLFOX therapy administration. The genomic signature of oxaliplatin exposure (G > T/C > A, T > A/A > T) was not enriched after FOLFOX chemotherapy. Overlapping single nucleotide variants (SNVs) and indels remained in 26–65% of the patient-matched tumor samples. One patient harbored an *AKT1* E17K mutation in the recurrent tumor, whereas *PIK3CA* E542K and E88Q mutations were detected in the primary and untreated metastatic tumor samples. Genes related to intracellular Ca^2+^ homeostasis were enriched among the genes uniquely mutated after FOLFOX chemotherapy.

**Conclusions:**

We found that the mutation rates, mutation spectrum, and copy number variants were nearly identical regardless of the administration of FOLFOX therapy in the four CRC cases. The mutational discordance between the patient-matched tumor samples is likely caused by tumor heterogeneity and chemotherapy-induced clonal selection. These findings might be useful as pilot data for larger studies to clarify the changes in the mutational landscape induced by adjuvant FOLFOX chemotherapy.

**Electronic supplementary material:**

The online version of this article (10.1186/s12885-019-5479-6) contains supplementary material, which is available to authorized users.

## Background

Colorectal cancer (CRC) is the third most common type of cancer and the fourth leading cause of cancer death worldwide [1]. Combination chemotherapy with cytotoxic and molecular targeting agents has prolonged the survival time of patients with metastatic CRC. In the era of genome-based personalized cancer treatment, an assessment of the molecular profile of individual tumors is necessary to guide the selection of appropriate therapy methods. For example, activating *KRAS* and *NRAS* mutations are negative predictive markers for the effectiveness of the anti-epidermal growth factor receptor (EGFR) antibodies panitumumab and cetuximab [2, 3]. Intensive chemotherapy is recommended for patients with the *BRAF* V600E mutation because this mutation is a strong prognostic factor for poor survival [4, 5]. Moreover, several genetic alterations that are potential prognostic and predictive biomarkers or therapeutic targets have been explored. Extensive data sets of the mutational profiles of CRC have been generated [6], and large collaborations have created gene expression-based classifications that predict patient outcomes [7].

However, systemic chemotherapies could alter the mutational landscape of several cancers [8, 9]. A previous exome sequencing study revealed that mutagenic chemotherapy regimens, such as adjuvant chemotherapy with the DNA-alkylating-like agent temozolomide to treat glioma, can induce new mutations and cause the malignant progression of recurrent tumors [9].

FOLFOX is a combination chemotherapy regimen that consists of leucovorin-modulated 5-fluorouracil (5-FU) and oxaliplatin (L-OHP), which are commonly used worldwide as standard adjuvant chemotherapies for curatively resected stage III and IV CRCs [10]. L-OHP is a third-generation platinum (Pt)-containing antitumor compound that induces DNA damage associated with intra- and inter-strand cross-links (Pt-GG adducts) [11–13]. Previously, in vitro studies have demonstrated the mutagenic activity of L-OHP [14]. Therefore, adjuvant FOLFOX chemotherapy has the potential to alter the mutational profiles of recurrent cancers so that they differ from those of primary CRC tumors. Our previous report showed that the mutational status of predictive biomarker genes for the effectiveness of anti-EGFR-antibodies was not altered by FOLFOX therapy [15]. However, the influence of FOLFOX therapy on exome-wide mutational profiles has not been reported previously.

This study used whole exome sequencing to compare gene alteration profiles of recurrent cancers after adjuvant FOLFOX chemotherapy in patient-matched primary CRC and metastatic tumor samples prior to chemotherapy.

## Methods

### Patient selection

We reviewed the clinical records of patients with CRC who had been treated with adjuvant FOLFOX chemotherapy after curative resection at the National Cancer Center Hospital East (Kashiwa, Japan). From January 2006 to December 2009, 156 patients were treated with adjuvant FOLFOX at our institution, and 66 patients developed recurrent tumors during or after adjuvant FOLFOX chemotherapy. Of these patients, 26 underwent curative resection of recurrent tumors. We selected four CRC patients for whom tumor specimens from primary tumors, metastases resected prior to FOLFOX chemotherapy (pre-FOLFOX metastasis), and recurrent metastatic tumors in the same organ after adjuvant FOLFOX (post-FOLFOX metastasis) were available. Informed consent to use tissue specimens for this study was obtained from all patients, and the tissue samples were provided by the National Cancer Center Biobank, Japan. The institutional review board at the National Cancer Center approved the study protocol. This study was performed according to the Epidemiological Study Guidelines of the Ministry of Health, Labor, and Welfare of Japan. We disclosed the study design on the National Cancer Center website and gave the relatives of deceased patients the opportunity to decline participation.

### DNA samples

We obtained matched primary CRC, pre-FOLFOX metastasis, post-FOLFOX metastasis, and normal colorectal tissue samples from four patients. The normal colorectal tissues were collected from surgical specimens of the primary tumors. All tissue samples were formalin-fixed, paraffin-embedded (FFPE) specimens. DNA samples were obtained from macroscopically dissected FFPE specimens cut into 10-μm-thick sections. Genomic DNA was extracted using EZ1 Advanced XL and EZ1 DNA Tissue Kits (Qiagen, Hilden, Germany) according to the manufacturer’s instructions [16]. Nucleic acid yields were determined using a NanoDrop 2000 (Thermo Fisher Scientific, Waltham, MA, USA), and the quality of genomic DNA was examined using a Quant-iT picoGreen dsDNA (Life Technologies, Carlsbad, CA, USA) assay kit.

### Whole exome sequencing and variant calling

Using genomic DNA from tumors and matched normal samples, we performed exome capture sequencing. Using an Agilent SureSelect Human All Exome V5 + UTRs kit (Agilent Technologies, Santa Clara, CA, USA), whole exome sequencing was performed using an Illumina HiSeq 2000 system (Illumina, San Diego, CA, USA) to generate 100-bp paired-end sequencing reads according to the manufacturer’s instructions. Burrows-Wheeler Aligner (BWA, http://bio-bwa.sourceforge.net/) [17] was used to align the sequencing reads to the human reference genome (hg19). The Genome Analysis ToolKit version 1.6 (GATK, http://www.broadinstitute.org/gatk/) was used for the local realignment and score recalibration of the sequencing reads [18]. We employed Picard (http://broadinstitute.github.io/picard/) for the basic processing and management of the sequencing data. To reduce the false-positive rate, the following filtering criteria were applied: (i) GATK confidence score [18] ≥ 50; (ii) number of forward and reverse reads ≥1; and (iii) variants present in at least 10% of the reads. All mutations detected in the paired non-tumor colon tissues were excluded from our analysis. We also excluded alterations present in dbSNP151, the 1000 Genomes Project, and in-house Japanese exomes derived from 299 normal tissues in our previous studies, with the aim of identifying tumor-specific variants. We also performed a visual inspection to filter out false-positive variants.

All mutations with clinical inference were annotated using ANNOVAR [19].

### **Gene ontology analysi**s

A Gene Ontology (GO) analysis was performed using the Database for Annotation, Visualization, and Integrated Discovery (DAVID, http://david.abcc.ncifcrf.gov) [20]. Adjusted *P*-values less than 0.05 were considered significant. The GO analysis was performed for nonsense mutations, small insertions/deletions (indels), and missense mutations that were predicted as “probably damaging” and “possibly damaging” by PolyPhen2 (http://genetics.bwh.harvard.edu/pph2/) [21].

### Copy number variant analysis

The log ratio of the depth of coverage between the tumor and normal colorectal tissues was calculated using the GATK-depth of coverage tool. Then, copy number variant (CNV) segments were identified from the log ratio of the depth of coverage using the Exome CNV R package [22]. Log ratios of the depth of coverage that were greater than two were considered indicative of significant copy number amplification.

### Statistical analysis

The Wilcoxon signed-rank test was used to evaluate the difference in the number of genetic alterations between primary, pre-FOLFOX metastatic, and post-FOLFOX metastatic tumors. Increases in specific mutation types among the post-FOLFOX unique mutations were also assessed using this method. Microsoft Office Excel 2013 (Microsoft Corporation, Redmond, WA, USA) was used to perform all the statistical analyses.

## Results

### Patient characteristics and clinical courses

Table 1 shows the patient characteristics. There were two male and two female participants with a median age of 68 years. The primary tumor sites were the colon in one patient and the rectum in three patients. After curative resection of their primary and metastatic tumors, all patients were treated with a modified FOLFOX6 (mFOLFOX6) regimen including an L-OHP dose of 85 mg/m^2^ administered every 14 days; 12 treatment cycles were planned [23]. Post-FOLFOX metastasis developed during (cases 1 and 2) or after (cases 3 and 4) adjuvant chemotherapy. Histopathological analyses diagnosed the primary CRC tumors as well-differentiated adenocarcinoma (cases 2 and 4) and moderately differentiated adenocarcinoma (cases 1 and 3). All metastatic tumors exhibited histology concordant with the corresponding primary colorectal adenocarcinoma.

**Table 1:** Patient characteristics

Case	Age range	Sex	Primary site	Histopathological type	Metastatic site (Pre−/Post-FOLFOX)	FOLFOX cycles	DFS (days)	Days from end of FOLFOX until recurrence
1	65–69	Male	S	Mode	Liver	4	97	−16^a^
2	65–69	Male	Rs-S	Well	Liver	9	109	−88^a^
3	60–64	Female	Rs	Mode	Liver	11	328	120
4	65–69	Female	Rb	Well	Lung	12	556	264

*S* sigmoid colon, *Rs* rectosigmoid, *Rb* rectum below the peritoneal reflection

*Mode* Moderately differentiated adenocarcinoma, *Well* Well-differentiated adenocarcinoma

*DFS* Disease-free survival

^a^FOLFOX administered after recurrence

Additional file 1 Figure S1 summarizes the clinical courses of these four patients. In case 1, adjuvant mFOLFOX6 was initiated after the colectomy for primary sigmoid colon cancer and hepatic resection was performed for the synchronous colorectal metastases. After three cycles of mFOLFOX6, recurrence in the remnant liver was examined by computed tomography (CT) imaging. In case 2, the patient underwent high anterior resection and liver metastasectomy. Early recurrence in the liver was identified by CT imaging after three cycles of adjuvant mFOLFOX6, which was continued according to the clinician’s discretion. In total, the patient received nine cycles of mFOLFOX6 before resection of the liver recurrence. In case 3, the patient was diagnosed with stage IIA rectal adenocarcinoma, received lower anterior resection without chemoradiotherapy, and was followed without adjuvant chemotherapy. Liver metastasis was diagnosed 14 months after the first operation. Adjuvant mFOLFOX6 was administered following liver metastasectomy, and adjuvant therapy was discontinued at 11 cycles as a result of intolerable peripheral sensory neuropathy. Liver recurrence was identified four months after the end of adjuvant chemotherapy. In case 4, the patient underwent lower anterior resection and lung metastasectomy for metastatic diseases. Nine months after completing the planned adjuvant chemotherapy, lung recurrence was identified by CT imaging.

A median of 10 mFOLFOX6 cycles was reported in this study (range, 4–12 cycles), and the median disease-free survival was 218.5 days (range, 97–556 days).

### Comparison of the mutation rates and analysis of the mutation spectrum

Whole exome sequencing was performed to investigate the profile of somatic alterations in all tumor samples. The tumors were sequenced to an average 124-fold coverage (range, 90–155), enabling the sensitive detection of single nucleotide variants (SNVs) and indels to a 10% variant frequency. To identify germline mutations, we sequenced the paired non-tumor colon tissues in addition to the tumor tissues from each patient. Additional file 2: Table S1 shows a summary of the coverage details.

**Fig. 1:**
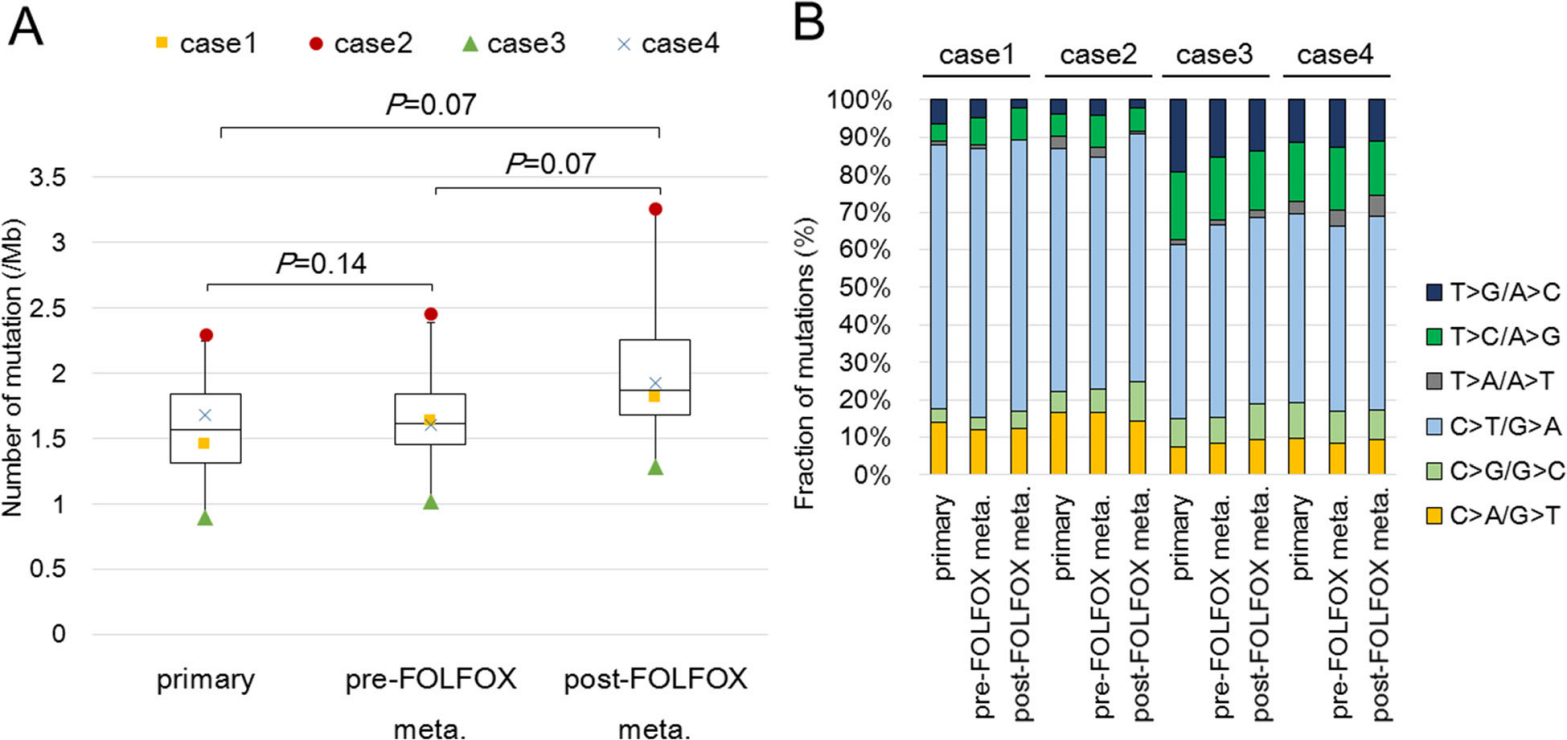
**a** Comparison of mutation rates: Box-and-whisker plot showing the range, median, and 25th and 75th percentile levels of the number of mutations in all primary, pre-FOLFOX metastatic, and post-FOLFOX metastatic tumor samples. The difference in the number of mutations was not significant among the primary, pre-FOLFOX metastatic, and post-FOLFOX metastatic tumor samples across all cases (*P* > 0.05, Wilcoxon signed-rank test) **b** Comparison of mutation spectra: Stacked bar plot showing the proportion of mutations accounted for by each of the six mutation types in all primary, pre-FOLFOX metastatic, and post-FOLFOX metastatic tumor samples for each case

The mutation rate of the somatic SNVs and indels in each tumor sample ranged from 0.90 to 3.26/Mb, with a median of 1.66/Mb. The rates were consistent with those of non-hypermutated CRC cases reported by The Cancer Genome Atlas (TCGA) [6]. Although the mutation rates increased slightly after FOLFOX administration, there were no significant differences between the matched primary CRC, pre-FOLFOX metastatic, and post-FOLFOX metastatic samples (*P* > 0.05, Fig. 1a).

**Fig. 2:**
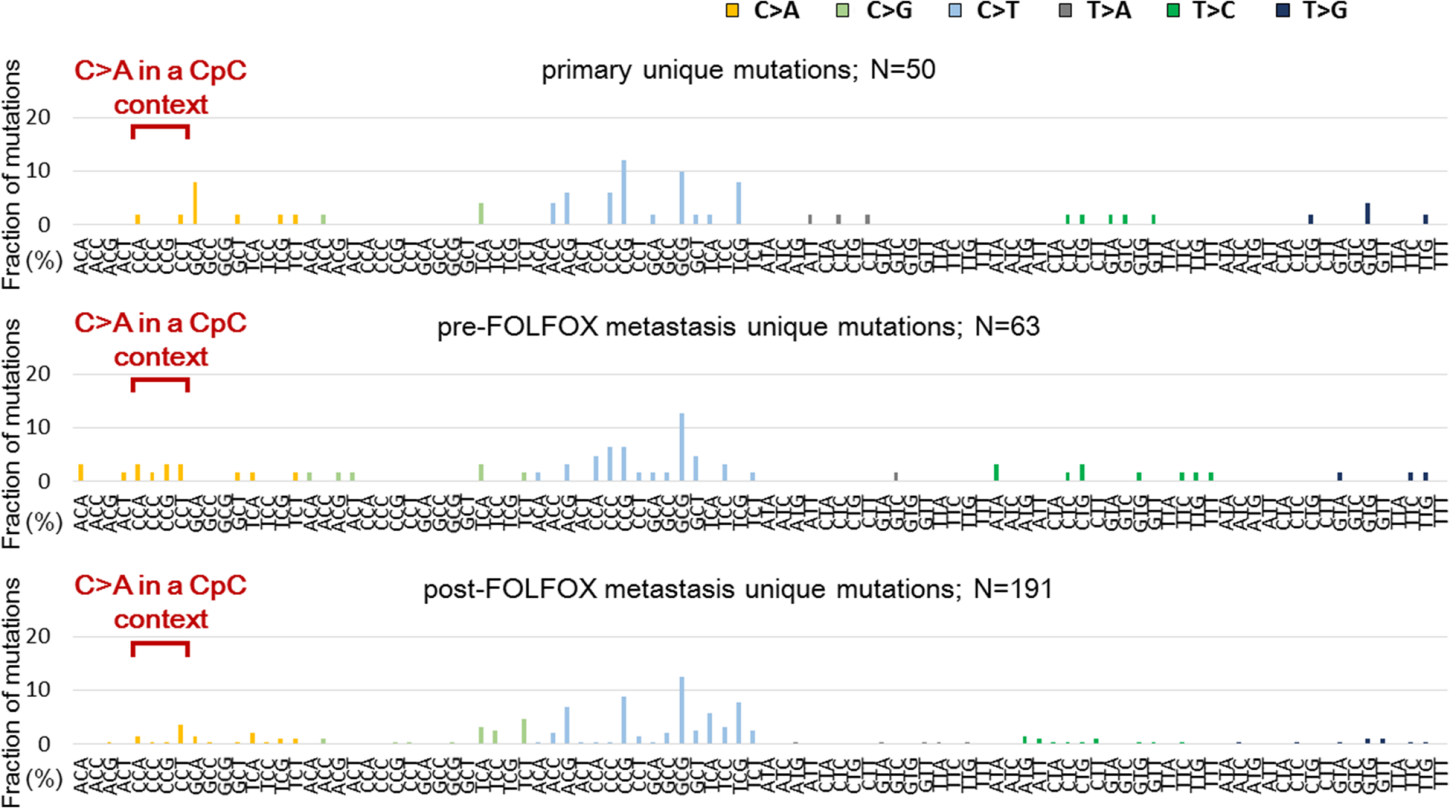
Comparison of the mutation spectrum among unique mutations: The relative frequencies of 96 trinucleotides are shown for unique mutations in the primary, pre-FOLFOX metastatic, and post-FOLFOX metastatic tumor samples. The presented number of mutations is the sum of the data recorded for the four patients. For specific mutation types, a Wilcoxon signed-rank test was used, and significant enrichment in the platinum exposure signature, C > A in a CpC context (equivalently, G > T in a GpG context), was not observed

Next, the mutation spectrum of each tumor was investigated. All tumor samples harbored a predominance of C > T/G > A transitions (range, 45–70%), which is reported as a mutational signature of CRC [6, 24] (Fig. 1b). C > A/G > T and T > A/A > T transversions were reported previously as the most common mutations produced by L-OHP in cultured cells [14]. In addition, C > A mutations were particularly prominent in a CpC context (equivalently, G > T in a GpG context) as a result of intrastrand cross-links induced by cisplatin, which is a Pt-containing antitumor compound similar to L-OHP [25]. Therefore, we evaluated whether these platinum exposure signature mutations were enriched among the post-FOLFOX unique mutations. Figure 2 shows the mutation spectra of the primary CRC, pre-FOLFOX, and post-FOLFOX unique mutations. There was no significant increase in C > A/G > T (primary vs post-FOLFOX metastasis, *P* = 0.27; pre- vs post-FOLFOX metastasis, *P* = 1.00) or T > A/A > T (primary vs post-FOLFOX metastasis, *P* = 1.00; pre- vs post-FOLFOX metastasis, *P* = 0.14) transversions among the post-FOLFOX unique mutations. The incidence of C > A mutations in a CpC context was not increased in post-FOLFOX metastasis (primary vs post-FOLFOX metastasis, *P* = 0.18; pre- vs post-FOLFOX metastasis, *P* = 0.18). In addition, there were no significant differences in other mutation fractions (Additional file 3: Figure S2).

### Analysis of CNVs

**Fig. 3:**
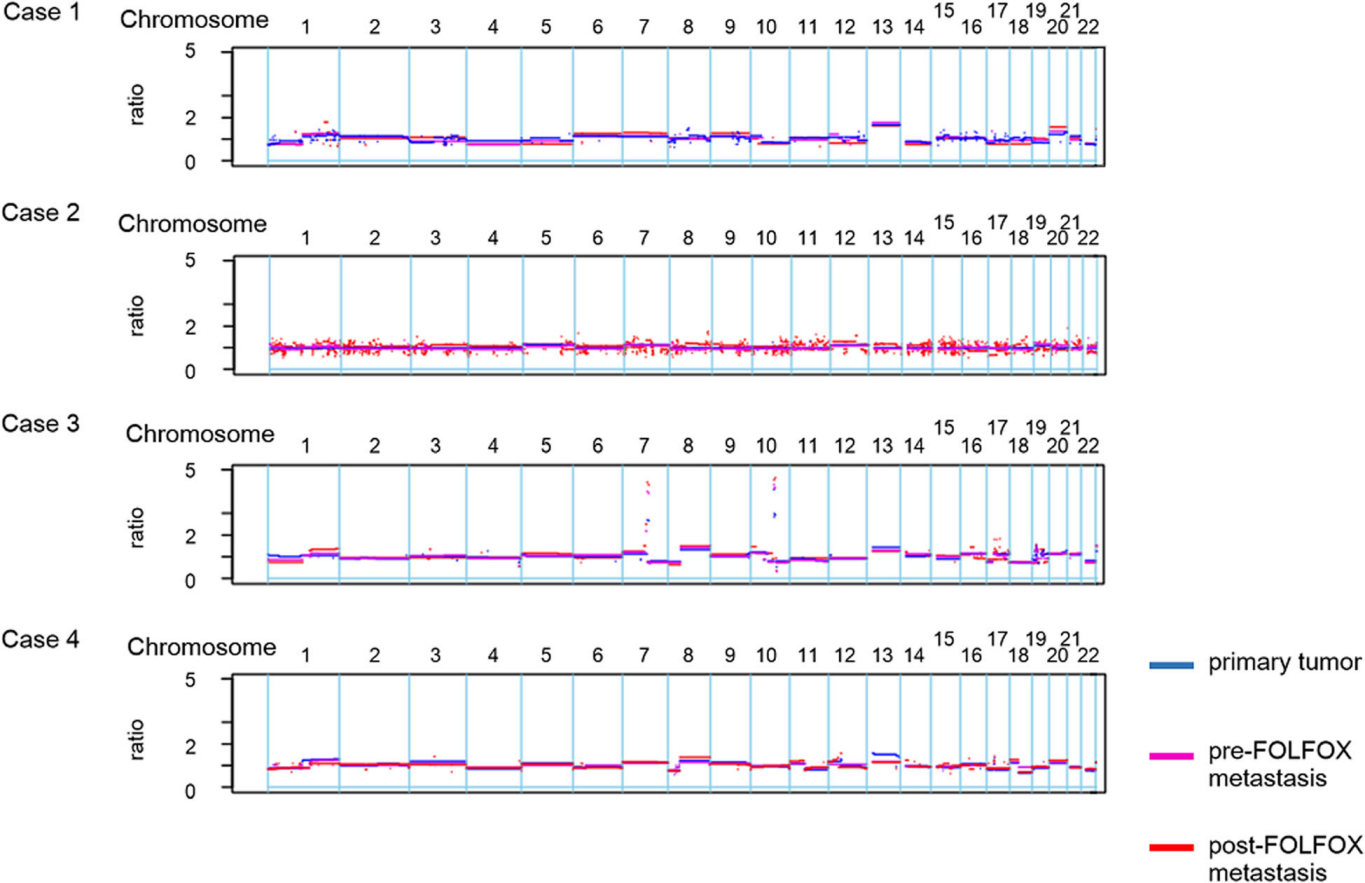
Comparison of copy number alterations: The CNV patterns were similar among primary (blue), pre-FOLFOX metastatic (purple), and post-FOLFOX metastatic (red) tumor samples in all cases. The x-axis coordinates represent positions along the genome; the vertical bars indicate the borders between chromosomes. The y-axis represents the chromosomal copy number in the tumor compared to normal tissues. A log ratio greater than two was considered significant copy number amplification

To evaluate whether the CNV was affected by FOLFOX treatment, the CNVs in post-FOLFOX metastatic tumors were compared to those in primary tumors and pre-FOLFOX metastatic tumors. Our comparative analysis revealed that CNVs persisted regardless of the administration of FOLFOX therapy in any tumor sample in cases 1, 2, and 4 (Fig. 3). In case 3, we observed focal amplification of the 7q21, 10q22, and 10q23 chromosomal regions, which persisted regardless of FOLFOX therapy administration. However, the amplification of some genes, such as *SEMA3E*, *SEMA3A*, *PCLO*, *AK055932*, and *BX647900*, was observed only in pre- and post-FOLFOX metastasis and not in the primary tumor in case 3 (Additional file 4: Table S2).

### Overlap of SNVs and indels between patient-matched tumor samples

**Fig. 4:**
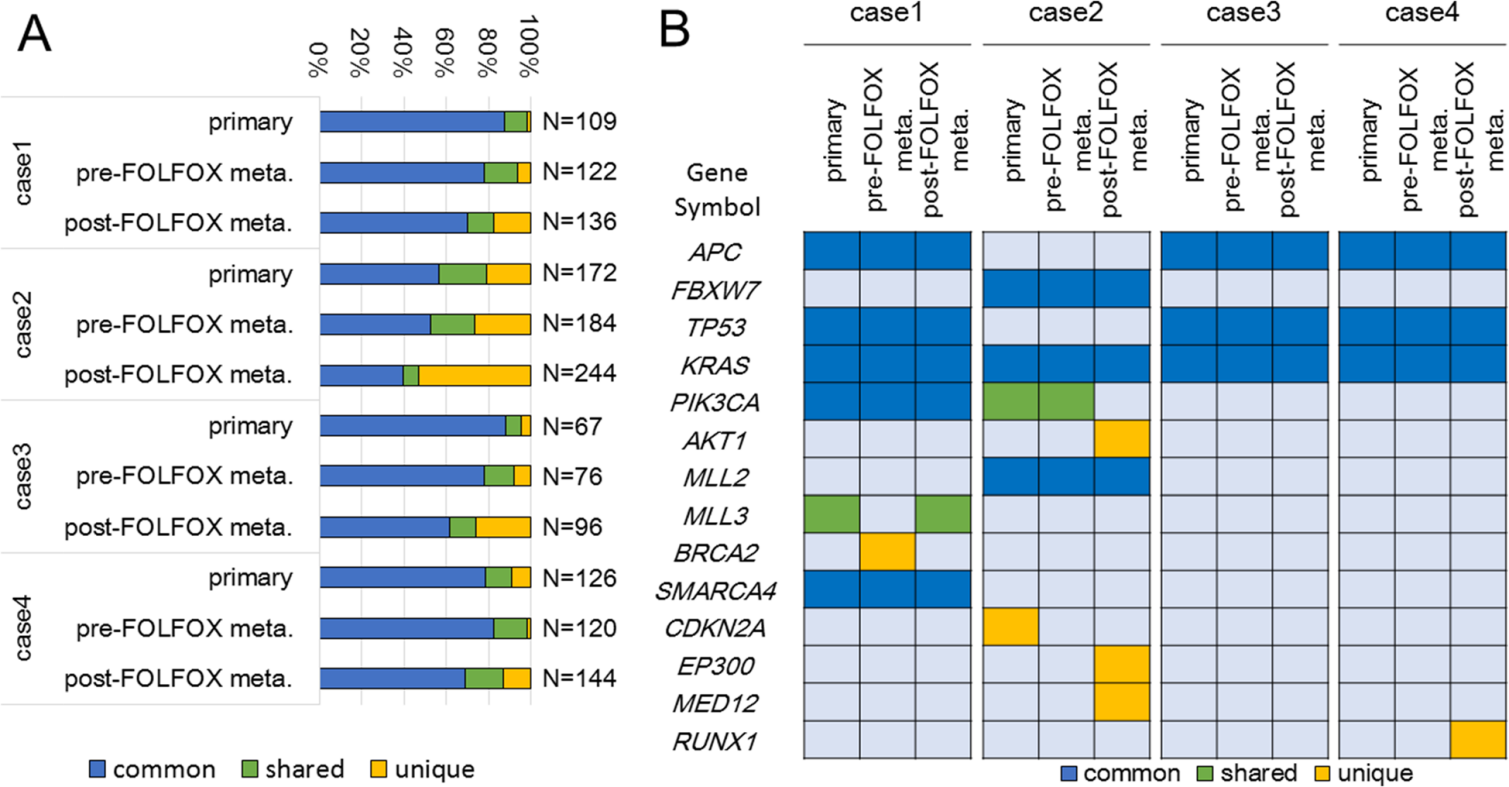
**a** Overlap of SNVs and indels: All somatic mutations and indels were classified into three categories: mutations present in all tumor regions (common, blue), mutations shared in more than two but not all regions (shared, green), and mutations in one tumor region (unique, yellow) in each case. The distributions of all somatic mutations in each tumor sample are shown in a stacked bar plot. The number of analyzed mutations is displayed on the right side of each bar. **b** Comparison of Mut-driver gene mutations: Mutations in 14 Mut-driver genes were detected in all tumor samples, and eight of these were classified as common mutations. A discordant mutation in the PI3K pathway before and after FOLFOX administration was observed in case 2

The overlap of the detected mutations between the primary, pre-FOLFOX metastatic, and post-FOLFOX metastatic tumor samples in the individual cases was investigated. Additional file 5: Table S3 lists the details of all detected gene mutations. Of the gene mutations detected in each post-FOLFOX metastatic sample, 112 (82%) in case 1, 114 (46%) in case 2, 71 (73%) in case 3, and 125 (86%) in case 4 were shared in the matched tumor samples, indicating that 14–54% of the mutations were post-FOLFOX unique mutations (Fig. 4a).

Next, we focused on Mut-driver genes, as advocated by Vogelstein et al. [26]; these genes include tumor suppressor genes for which at least 20% of the mutations caused truncation of the gene product and oncogenes for which at least 20% of the missense mutations occurred at a single position along the polypeptide chain. High mutational concordance of these genes, including frequent mutation in CRC genes such as *APC*, *KRAS*, *FBXW7*, *TP53*, and *PIK3CA*, was observed between the matched tumor samples in the individual cases (Fig. 4b). However, in case 2, a lack of *PIK3CA* mutations was found in the post-FOLFOX metastatic samples, although the *PIK3CA* E542K and E88Q mutations were detected in both the primary tumor and pre-FOLFOX metastatic samples. By contrast, *AKT1* E17K (C > T) mutations were found only in the post-FOLFOX metastasis samples in case 2.

After FOLFOX administration, we also identified the gain or loss of some mutations that were predicted as functionally important variants by Polyphen2 [21]. Additional file 6: Table S4 lists the details of these mutations.

### Gene ontology analysis of post-FOLFOX unique mutations

Post-FOLFOX unique mutations in recurrent tumors may reflect the mechanism of chemo-resistance because recurrent tumors are thought to develop through chemotherapy-induced selective pressure. A GO analysis was performed to identify the molecular functions enriched in post-FOLFOX metastatic samples. For this analysis, we selected gene mutations that predict a possible impact of an amino acid substitution on the structure and function of a human protein (the analyzed gene lists are shown in Additional file 7: Table S5). Although no identical gene mutations were detected in more than two cases, four significant functional clusters were identified (Table 2). The genes annotated to the GO term “calcium ion binding” were detected in all cases, and genes annotated to “calcium ion transport” were detected in three cases. These GO terms were not enriched among the pre-FOLFOX unique gene mutations or among common mutations (Additional file 8: Table S6).

**Table 2:** Significant functional clusters among post-FOLFOX unique mutations

Gene Ontology term	Gene symbol				Adjusted *P* value
	Case 1	Case 2	Case 3	Case 4	
GO:0007155~cell adhesion	*–*	*CDH9* *TRPM7* *COL22A1* *NLGN4X* *PCDH17*	*PCDHA9* *PDZD2*	*–*	0.033
GO:0030695~GTPase regulator activity	*–*	*DOCK1* *TBC1D9* *RICTOR* *IQSEC1*	*–*	*TBC1D12*	0.037
GO:0006816~calcium ion transport	*CACNA1E*	*TRPM7* *TRPV5* *SLC24A6*	*–*	*JPH3*	0.0014
GO:0005509~calcium ion binding	*CACNA1E*	*CDH9* *TRPM7* *TBC1D9* *TRPV5* *SLC24A6* *PCDH17*	*PDCHA9* *PLCB3*	*DOC2A* *RUNX1*	0.00044

## Discussion

In this study, we performed whole exome sequencing on primary colorectal, metastatic, and recurrent tumor samples after adjuvant FOLFOX therapy to evaluate the influence of this cytotoxic chemotherapy regimen on the mutational profile of recurrent CRC. Recently, a wide range of genomic alterations have displayed associations with cancer behavior, and most of these alterations are typically found in coding regions [27]. Therefore, whole exome sequencing is a reasonable strategy for identifying clinically actionable alterations induced by adjuvant FOLFOX chemotherapy in recurrent CRC. Furthermore, one of the most important aims of this study was to evaluate whether adjuvant FOLFOX chemotherapy produces genetic alterations in recurrent CRC. Thus, we excluded variants that could possibly be regarded as germline alterations from our analysis by using the sequencing results from patient-matched normal tissues and previous SNP databases.

Our data showed that the mutation rates and mutation spectrum were nearly identical between the primary CRC, pre-FOLFOX metastatic, and post-FOLFOX metastatic samples. Although the mutagenic activity of L-OHP was demonstrated in cultured cells [14], an enrichment in the L-OHP signature was not observed in post-FOLFOX metastatic tumors. Differences between the results of that study and our results may be due to differences in the drug concentration used in that in vitro study and the concentration used in clinical practice. A significant dose-dependent increase in mutation frequency was observed when CHO-K1 cells were exposed to 10–40 μM L-OHP [14]. However, the Cmax of L-OHP has been reported as approximately 3.6 μM in the clinical dose setting with mFOLFOX6 therapy (85 mg/m^2^) [28], which might explain the differences between the in vitro findings and those of the present study. A recent study revealed that some recurrent gliomas were hypermutated and harbored driver mutations in the RB and Akt-mTOR pathways that bore the signature of temozolomide-induced mutagenesis after adjuvant temozolomide chemotherapy. Recurrent gliomas that showed evidence of temozolomide-induced hypermutation underwent malignant progression to high-grade tumors with a poorer prognosis [9]. By contrast, our study suggests that FOLFOX is a safe regimen that lacks the potential risk of inducing new driver mutations and malignant progression, unlike adjuvant chemotherapy involving temozolomide to treat glioma.

The patterns of CNVs were nearly identical before and after FOLFOX chemotherapy in all the cases. The focal amplification of the 7q21, 10q22, and 10q23 chromosomal regions in the post-FOLFOX metastasis samples was also detected in the primary or pre-FOLFOX metastasis samples in case 3. However, we revealed that some gene amplifications were observed only in the pre- and post-FOLFOX metastasis samples and not in the primary tumor samples in case 3. Among these genes, we focused on *SEMA3E*, which has an expression level reported to be positively correlated with increased metastasis in ovarian, melanoma, and colon cancers [29]. Our results suggest that the acquisition of *SEMA3E* gene amplification might relate to the metastatic potential in CRC and that CNVs can change during the process of tumor progression.

One of the limitations of our study is its small sample size due to the strict patient selection criteria. Several studies have reported that differences in mutational profiles depend on the metastatic site [30–32]. Therefore, it was necessary to analyze patient- and organ-matched pre-FOLFOX metastasis and post-FOLFOX metastasis samples. However, such samples are rarely found in daily clinical practice. Our analysis used valuable samples from four CRC cases and accurately assessed the influence of adjuvant FOLFOX chemotherapy on the mutational profile of CRC. The other limitation is the possibility that our cases may have had primary resistance to FOLFOX therapy. This resistance may have been why we failed to observe a chemotherapy-induced signature in the recurrent tumor samples. Our findings should be further validated in a large cohort that includes multiple patients with various backgrounds, such as variations in the duration of FOLFOX chemotherapy or the timing of recurrence.

It is also possible that there was simply not enough time to observe a chemotherapy-induced signature in the recurrent tumor samples that we used. Twelve-cycle FOLFOX chemotherapy was planned as the full adjuvant chemotherapy course, but it was often discontinued due to tumor relapse or intolerable adverse events. Our data reflect a correlation between adjuvant FOLFOX chemotherapy and the mutation profile of CRC in clinical practice, but further investigation is necessary regarding whether long-term treatment with FOLFOX chemotherapy, such as chemotherapy for unresectable CRC, can induce additional mutations. A previous study suggested that a circulating tumor DNA analysis could be used to reliably monitor tumor dynamics in subjects with cancer who were undergoing surgery or chemotherapy for CRC [33]. This non-invasive approach may be a more suitable way to test residual tumor cells and analyze temporal changes in mutation profiles.

Previous comparative genomic studies have reported varying degrees of divergence in the genomic profiles of primary CRC and matched metastases. Substantial mutational divergence between paired primary tumor and metastasis samples was reported in a whole exome sequencing analysis with a low sequencing depth [34] and in shallow-targeted sequencing analysis variants with a variant allele frequency greater than 20% [35]. By contrast, a recent deep-targeted sequencing analysis [36, 37] and whole exome sequencing study with an average sequencing depth of > 100-fold [38] showed a high degree of mutational concordance, generally 50–80%, between primary CRC and metastases. These studies also suggest an exceedingly high level of concordance between primary CRC and metastases in genetic alterations that occur early in colorectal carcinogenesis, such as alterations in *APC*, *KRAS*, *NRAS*, and *BRAF*. In our study, we achieved an average coverage depth of 124-fold, enabling the sensitive detection of SNVs and indels to a 10% variant frequency. Our results are comparable to the results of previous high-resolution sequencing analyses. Therefore, it is more accurate to state that the majority of mutations are shared between primary CRC and metastatic tumors in at least some cases. Furthermore, our finding confirms our previous results [15] showing that both primary tumors and their subsequent metastases could be valid sources of DNA for patient selection before commencing anti-EGFR therapy.

Brannon et al. demonstrated that some of the mutational discordance could be explained by spatial heterogeneity in the patient tumors. They also suggest chemotherapy-derived clonal selection based on the finding that pre-treated tumors were less likely to have unique mutations than chemo-naive tumors in patients with concurrently resected tumors [36]. The increase in unique mutations after FOLFOX chemotherapy in our cases is also likely explained by intra-tumor heterogeneity and clonal selection rather than by the mutagenicity of L-OHP. According to the Big Bang model of human colorectal tumor growth [39], numerous heterogeneous subclones predominantly expand through tumor evolution. After treatment-derived clonal selection via methods such as surgery and cytotoxic chemotherapy, clones acquiring a fitness advantage in the environment become dominant in residual tumors. Our findings raise the possibility that surgery and adjuvant FOLFOX chemotherapy change the clonal composition, resulting in differences in genetic alterations before and after FOLFOX administration. This hypothesis may explain the mutational discordance in case 2. The *AKT1* E17K mutation can activate the PI3K/AKT/mTOR pathway in a similar manner as *PIK3CA* alterations and promotes carcinogenesis through increasing cell proliferation or survival [40] via the PI3K pathway. *AKT1* mutant clones, which were found in the minority of primary tumor samples, might sufficiently increase via clonal selection to detectable levels following surgery and adjuvant chemotherapy. The high rate of unique mutations in post-FOLFOX metastatic samples (54% in case 2) also indicates that there are differences in the clonal composition of these tumors compared to those of primary tumor and pre-FOLFOX metastatic samples. However, because of the very limited number of cases and the heterogeneity of the tumors, further validation is necessary. Furthermore, in a recent report, Angelova M et al. demonstrated that the immune system also influences tumor clonal composition and tumor evolution during the metastatic process [41]. In future studies, we must focus on clonal selection induced by not only chemotherapeutic agents but also by the immune system.

In addition, we reported that some genes that might be related to drug resistance were gained or lost during chemotherapy. Previous studies have indicated that *CIAPIN1* is involved in the development of multiple drug resistance (MDR) [42]. PTPRJ is expressed in CRC cells, and it is reported that the sustained inhibition of PTPRJ increased cell resistance to 5-fluorouracil (5-FU)-induced apoptosis [43]. Wang et al. indicated that the TopBP1 expression level was related to the prognosis of non-small cell lung cancer patients treated with platinum-based chemotherapy [44]. The *CIAPIN1* R132W and *PTPRJ* L738 V mutations were identified among the post-FOLFOX unique mutations in case 2. Conversely, in case 2, the *TOPBP1* S630 L mutation was identified in primary and pre-FOLFOX metastasis but not in post-FOLFOX metastasis. These mutations were non-synonymous and were predicted as functionally important variants by ANNOVAR [19] in addition to Polyphen2 [21]. Thus, these mutations may affect the function of proteins related to drug resistance. However, the true function of these mutations is unclear, and further investigation is necessary.

The results of the GO analysis showed an enrichment of genes that are annotated as “calcium ion transport” among genes uniquely mutated after FOLFOX. For example, *TRPM7* encodes a calcium-permeant ion channel that is notable for its inherent serine/threonine kinase activity [45]. The product of *CACNA1E* is the alpha 1E subunit of R-type voltage-dependent calcium channels [46]. The calcium-selective channel encoded by *TRPV5* is activated by a low internal calcium level [47]. *SLC24A6* encodes a family of potassium-dependent sodium/calcium exchangers that maintain cellular calcium homeostasis [48]. Previous in vitro studies have indicated that there is a relationship between intracellular Ca^2+^ homeostasis and the P-glycoprotein-dependent MDR phenotype [49], which is considered one of the mechanisms underlying resistance to L-OHP [50]. Although further investigation of the true function of these mutations is necessary in a larger cohort, alterations in genes involved in intracellular Ca^2+^ homeostasis may be related to resistance to FOLFOX therapy with the development of MDR.

## Conclusions

In conclusion, our data showed that the mutation rates, mutation spectra, or CNVs were nearly identical between the primary tumor, pre-FOLFOX metastatic, and post-FOLFOX metastatic samples in four CRC cases. We found that some gene mutations that might be related to FOLFOX resistance were gained or lost during chemotherapy, and the inter-tumor discordance of the mutational profiles suggests the existence of intra-tumor heterogeneity and the induction of clonal selection as a result of response to the FOLFOX chemotherapy. Our findings might be useful as pilot data for a larger study to clarify the changes in the mutational landscape induced by adjuvant FOLFOX chemotherapy.

## Additional files

Additional file 1: **Figure S1.** Clinical courses of the four cases. The blue arrows indicate the day of surgery, and the red arrows indicate the day on which the recurrent tumors were diagnosed during or after adjuvant FOLFOX therapy. The arrowheads (light blue) indicate the number of FOLFOX treatments. Disease-free survival (DFS) is calculated from the time of the final operation until post-FOLFOX recurrence (TIF 259 kb)

Additional file 2: **Table S1.** Coverage details. Summary of the sequencing coverage depth in each sample. (XLSX 10 kb)

Additional file 3: **Figure S2.** Comparison of the mutation spectrum among unique mutations. Relative mutation frequencies are shown for unique mutations in primary, pre-FOLFOX metastatic, and post-FOLFOX metastatic tumor samples. The presented number of mutations is the sum of data recorded in the four patients. There were no significant differences in all mutation fractions (Wilcoxon signed-rank test). (TIF 119 kb)

Additional file 4: **Table S2.** Focal amplification in case 3. Summary of the gene amplification in case 3. (XLSX 10 kb)

Additional file 5: **Table S3.** Detailed information for all detected gene mutations. List of all gene mutations detected in our analysis. (XLSX 86 kb)

Additional file 6: **Table S4.** The gain or loss of the mutations that were predicted as functionally important. (XLSX 13 kb)

Additional file 7: **Table S5.** Post-FOLFOX unique gene mutations used for the GO analysis. List of selected gene mutations for the GO analysis. (XLSX 16 kb)

Additional file 8: **Table S6.** Significant functional clusters in each mutation category. List of the Gene Ontology terms enriched among unique gene mutations before FOLFOX administration or among common mutations. (XLSX 10 kb)

## Abbreviations

5-FU: 5-fluorouracil
BWA: Burrows-Wheeler Aligner
CNV: Copy number variant
CRC: Colorectal cancer
CT: Computed tomography
DAVID: Database for Annotation, Visualization, and Integrated Discovery
EGFR: Epidermal growth factor receptor
FFPE: Formalin-fixed, paraffin-embedded
GATK: Genome Analysis ToolKit
GO: Gene Ontology
Indels: Small insertions/deletions
L-OHP: Oxaliplatin
MDR: Multi-drug resistance
Pt: Platinum
SNV: Single nucleotide variant
