# Supplementary figures and images for: Comparative sequence analysis of patient-matched primary colorectal cancer, metastatic, and recurrent metastatic tumors after adjuvant FOLFOX chemotherapy
Source: BMC Cancer. 2019 Mar 21;19:255. doi: 10.1186/s12885-019-5479-6 (PMC6429751; doi:10.1186/s12885-019-5479-6)

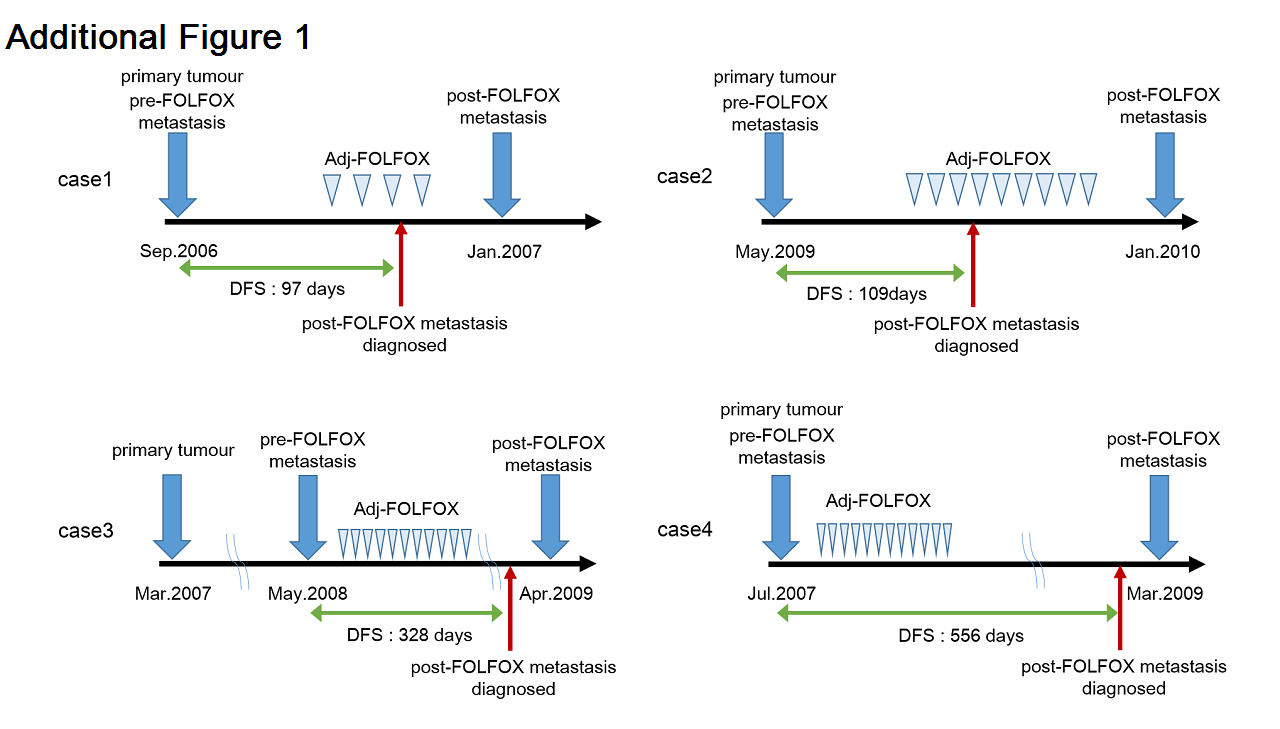

Supplement: Supplementary file 1 — Figure S1. Clinical courses of the four cases. The blue arrows indicate the day of surgery, and the red arrows indicate the day on which the recurrent tumors were diagnosed during or after adjuvant FOLFOX therapy. The arrowheads (light blue) indicate the number of FOLFOX treatments. Disease-free survival (DFS) is calculated from the time of the final operation until post-FOLFOX recurrence (TIF 259 kb) [file 12885_2019_5479_MOESM1_ESM.tif]

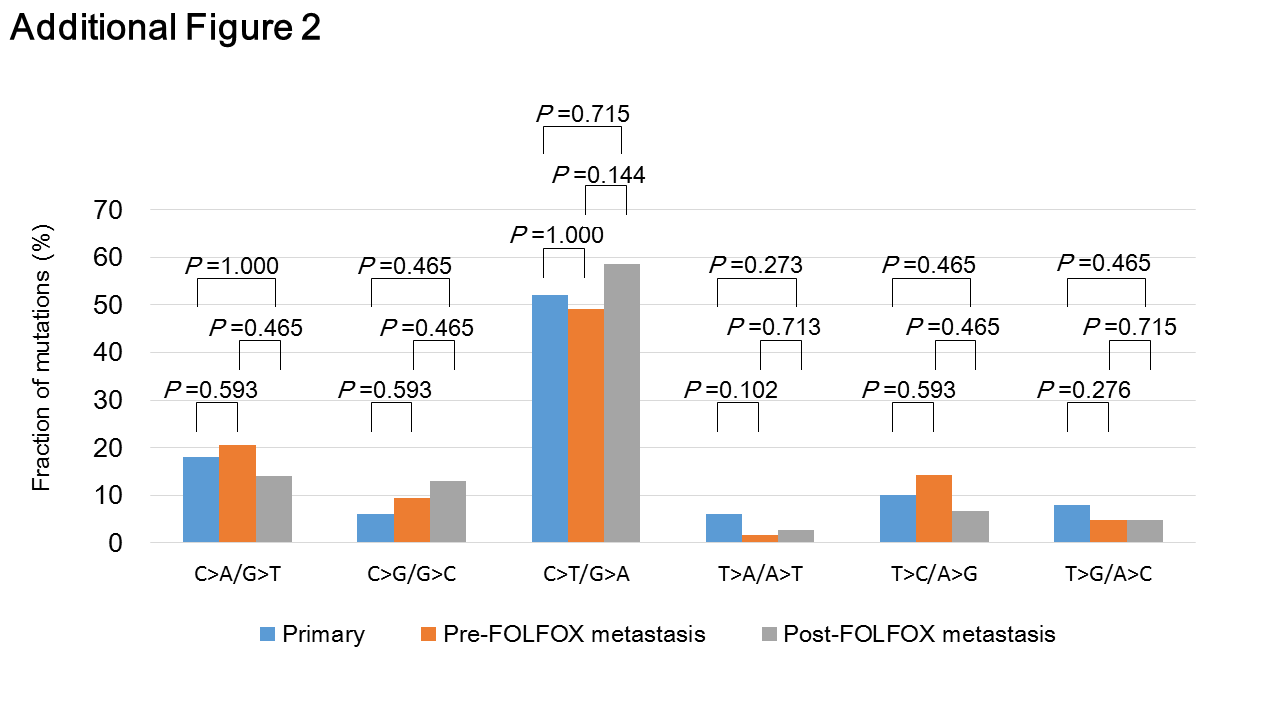

Supplement: Supplementary file 3 — Figure S2. Comparison of the mutation spectrum among unique mutations. Relative mutation frequencies are shown for unique mutations in primary, pre-FOLFOX metastatic, and post-FOLFOX metastatic tumor samples. The presented number of mutations is the sum of data recorded in the four patients. There were no significant differences in all mutation fractions (Wilcoxon signed-rank test). (TIF 119 kb) [file 12885_2019_5479_MOESM3_ESM.tif]
